# CAR-engineered NK cells; a promising therapeutic option for treatment of hematological malignancies

**DOI:** 10.1186/s13287-021-02462-y

**Published:** 2021-07-02

**Authors:** Faroogh Marofi, Marwan Mahmood Saleh, Heshu Sulaiman Rahman, Wanich Suksatan, Moaed E. Al-Gazally, Walid Kamal Abdelbasset, Lakshmi Thangavelu, Alexei Valerievich Yumashev, Ali Hassanzadeh, Mahboubeh Yazdanifar, Roza Motavalli, Yashwant Pathak, Adel Naimi, Behzad Baradaran, Marzieh Nikoo, Farhad Motavalli Khiavi

**Affiliations:** 1 Immunology Research Center (IRC), Tabriz University of Medical Sciences, Tabriz, Iran; 2 Department of Biophysics, College of Applied Science, University of Anbar, Ramadi, Iraq; 3 College of Medicine, University of Sulaimani, Sulaymaniyah, Iraq; 4 Department of Medical Laboratory Sciences, Komar University of Science and Technology, Chaq-Chaq Qularaise, Sulaimaniyah, Iraq; 5 Faculty of Nursing, HRH Princess Chulabhorn College of Medical Science, Chulabhorn Royal Academy, Bangkok, 10210 Thailand; 6 College of Medicine, Al-Ameed University, Karbala, Iraq; 7 Department of Health and Rehabilitation Sciences, College of Applied Medical Sciences, Prince Sattam bin Abdulaziz University, Al Kharj, Saudi Arabia; 8 Department of Physical Therapy, Kasr Al-Aini Hospital, Cairo University, Giza, Egypt; 9 Department of Pharmacology, Saveetha Dental College and Hospital, Saveetha Institute of Medical and Technical Sciences, Saveetha University, Chennai, India; 10 Sechenov First Moscow State Medical University, Moscow, Russia; 11 Department of Applied Cell Sciences, School of Advanced Technologies in Medicine, Tehran University of Medical Sciences, Tehran, Iran; 12 Stem Cell Transplantation and Regenerative Medicine, Department of Pediatrics, Stanford University School of Medicine, Palo Alto, CA USA; 13 Stem Cell Research Center, Tabriz University of Medical Sciences, Tabriz, Iran; 14 Professor and Associate Dean for Faculty Affairs, Taneja College of Pharmacy, University of South Florida, Tampa, FL USA; 15 Faculty of Pharmacy, Airlangga University, Surabaya, Indonesia; 16 Cellular and Molecular Research Center, Sabzevar University of Medical Sciences, Sabzevar, Iran; 17 Department of Immunology, School of Medicine, Kermanshah University of Medical Sciences, Kermanshah, Iran; 18 Department of Virology, Pasteur Institute of Iran, Tehran, Iran

**Keywords:** Natural killer (NK) cells, Chimeric antigen receptor (CAR), Immunotherapy, Hematological malignancies

## Abstract

Adoptive cell therapy has received a great deal of interest in the treatment of advanced cancers that are resistant to traditional therapy. The tremendous success of chimeric antigen receptor (CAR)-engineered T (CAR-T) cells in the treatment of cancer, especially hematological cancers, has exposed CAR’s potential. However, the toxicity and significant limitations of CAR-T cell immunotherapy prompted research into other immune cells as potential candidates for CAR engineering. NK cells are a major component of the innate immune system, especially for tumor immunosurveillance. They have a higher propensity for immunotherapy in hematologic malignancies because they can detect and eliminate cancerous cells more effectively. In comparison to CAR-T cells, CAR-NK cells can be prepared from allogeneic donors and are safer with a lower chance of cytokine release syndrome and graft-versus-host disease, as well as being a more efficient antitumor activity with high efficiency for off-the-shelf production. Moreover, CAR-NK cells may be modified to target various antigens while also increasing their expansion and survival in vivo. Extensive preclinical research has shown that NK cells can be effectively engineered to express CARs with substantial cytotoxic activity against both hematological and solid tumors, establishing evidence for potential clinical trials of CAR-NK cells. In this review, we discuss recent advances in CAR-NK cell engineering in a variety of hematological malignancies, as well as the main challenges that influence the outcomes of CAR-NK cell-based tumor immunotherapies.

## Introduction

In recent years, adoptive cell therapy has made unprecedented advances in the treatment of many tumors to conventional therapy based on the intrinsic properties of transplanted effector cells to target specific tumor antigens and remove tumors [1, 2]. A recent tremendous advance is based on chimeric antigen receptor (CAR)-engineered immune effector cells which improved cytotoxic activity of immune cells in the defense against tumor cells [3]. CARs are synthetic proteins consisting of a single-chain variable fragment (scFv) as an extracellular antigen-binding domain, connected to a diverse range of feasible intracellular activating signaling domain(s) that are designed to present a new ability to affect cells in the recognition of specific antigens in tumor cells and the final destruction of tumor cells [3, 4]. CAR is also a strategy that enables modified T cells to recognize a variety of MHC-independent tumor antigens. Autologous human T cells were the pioneer cells used in CAR therapy and have made a significant breakthrough in the treatment of patients with hematological malignant tumors, including chronic lymphocytic leukemia (CLL), and acute lymphoblastic leukemia (ALL), Hodgkin’s lymphoma, and non-Hodgkin’s lymphoma, and other B lymphomas [5–11].

Although CAR-modified T cell immunotherapy has exhibited great treatment effectiveness against hematological malignancies, several obstacles restrict their further clinical application [12, 13]. It is logistically challenging to generate an autologous CAR-T cell product separately for each patient, and CAR-T cell therapy economically is unfeasible for general clinical systems. Besides, several weeks are required for the production of CAR-T cells, which results in unavoidable inefficiency in the treatment of patients with aggressive diseases. Allogeneic T cells could be able to resolve these obstacles, but their transfer even if human leukocyte antigen (HLA) is matched between donors and recipients may pose a risk of severe graft-versus-host disease (GVHD) due to minor histocompatibility of antigens with genetic polymorphism of cytokines [14, 15]. Furthermore, the expansion and long-term persistence of CAR-T cells in the human body may cause serious and/or long-term adverse effects, such as cytokine release syndrome (CRS), which may threaten patients’ life in the process of secretion of pro-inflammatory cytokines [16, 17]. Natural killer cells have been demonstrated to prevail over the above drawbacks and provide promising alternative therapeutic targets for CAR engineering in adoptive immunotherapy since they do not require prior antigen sensitization or HLA matching, so they have the potential to be used as an allogeneic off-the-shelf product in cellular therapy and have minimal or no ability to induce graft-versus-host disease (GvHD) [18–20].

**Fig. 1:**
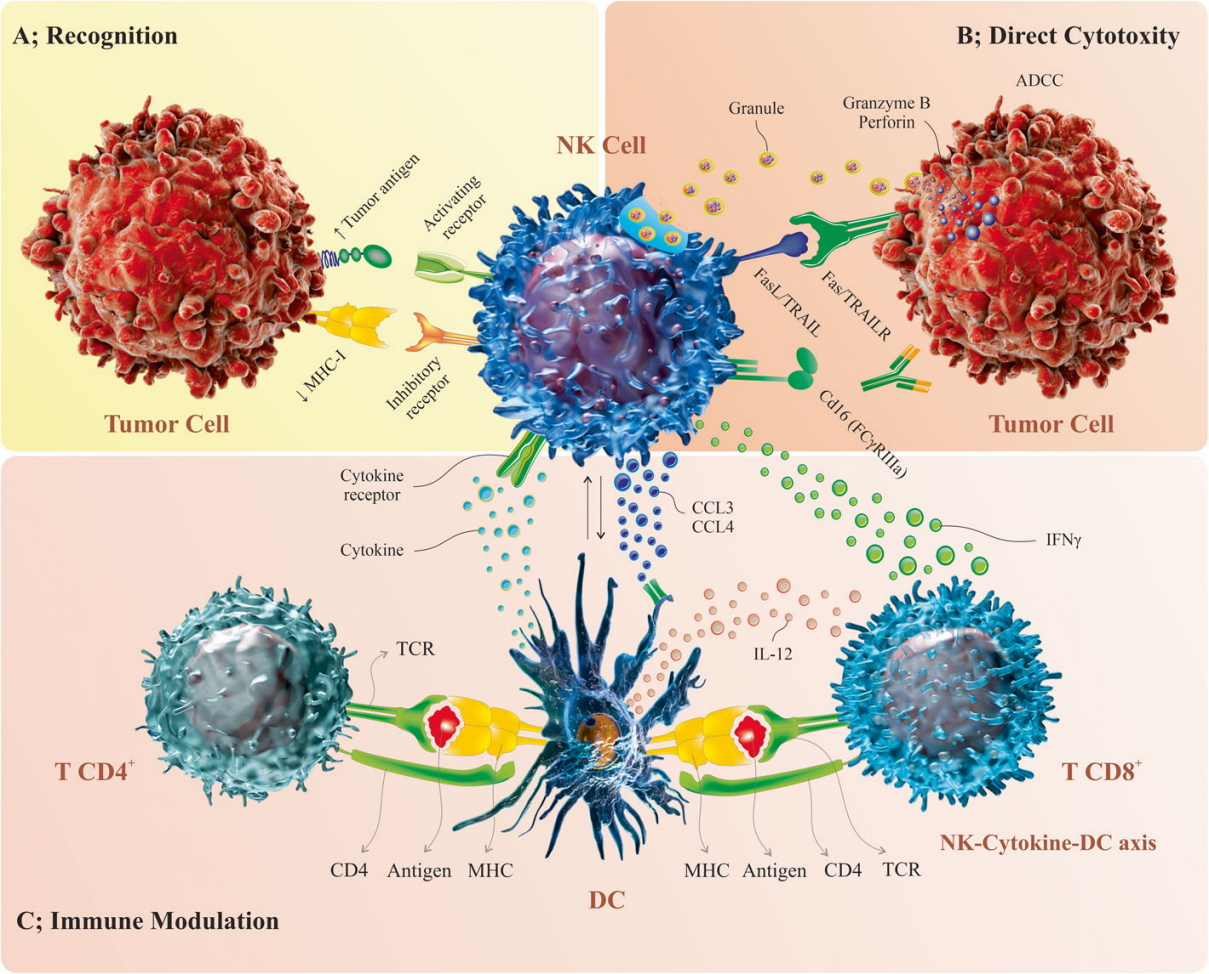
The NK cell-mediated cytotoxicity against tumor cells by affecting both the innate and adaptive immunity. As the innate killer cells, NK cells can identify the stress-induced ligands and reduced MHC I expression rates by own activating and inhibitory receptors, respectively (**A**). Upon activation, NK cells can stimulate the elimination of the tumor cells by the release of granules with granzyme B and perforin, the death receptor/ligand interaction and also ADCC (**B**). Further, activated NK cells can produce a spectrum of cytokine and chemokine that trigger the DC maturation and recruitment leading to the modulation of T cell response, more importantly the cytotoxic CD8+ T cell and Th cell induction (**C**). Natural killer (NK) cell, antibody-dependent cell cytotoxicity (ADCC), TNF-related apoptosis-inducing ligand (TRAIL), T helper (Th) cell, dendritic cell (DC)

Natural killer (NK) cells were discovered almost 50 years ago and are the first series of cytotoxic lymphocyte cells to defend against tumors in most tissues [21–23]. The natural function of NK cells is spontaneously triggered by specific stimulation and is ordered by the integration of signals from multiple germs line-encoded activating and inhibitory receptors which bind to homologous ligands in target tumor cells, assisting NK cells to identify malignant cells from normal cells. Then, NK cells can eliminate tumor cells directly and acutely through various pathways (Fig. 1) [22, 24, 25]. However, there are mechanisms by which the tumor escapes from the immune surveillance and inhibit the function of NK cells such as tumor microenvironment and immunosuppressive factors that prevent the expression of activating receptors and the interactions NK cells with other cells, also antigen escape ways can trigger inhibitory NK cell receptors and inhibit activating NK cell receptors [26–30]. To overcome these mechanisms, scientists have applied strategies directed at boosting the antitumor properties of NK cells and preventing immune escape through genetic engineering and the development of CAR-engineered NK cells [18, 31, 32]. Herein, we will discuss the advantages of CAR-NK cell immunotherapy and the challenges faced by CAR-NK cells, which must be removed before CAR-NK cell therapy replaces previous strategies, and then focuses on opportunities that highlight the efficacy of CAR-modified NK cells from preclinical studies in hematological malignancies.

## NK cell biology and adoptive tumor immunotherapy with NK cells

NK cells are critical cells of the innate immune system with the ability to perform cytotoxicity functions against tumor and virally infected cells quickly, spontaneously, and efficaciously without any previous sensitization, making them a desirable candidate for cancer immunotherapy [33–35]. The level of expression of the CD56 antigen surface, tissue location, and immune functions distinguish two main subgroups of NK cells: CD56bright cells and CD56dim cells [36, 37]. The CD56dim cells are fully mature and are the most common NK subgroup found in the peripheral blood and spleen. These cells predominantly have an immediate natural cytotoxic immune response to infected and also tumor cells. Furthermore, CD56dim cells express the CD16 (FcRIII) receptor, which is responsible for antibody-dependent cell-mediated cytotoxicity against antibody-opsonized tumor or virus-infected cells (ADCC) [38]. In contrast to CD56dim cells, CD56bright cells are more immature, have the maximum reproductive potential, are found in fewer subgroups in the peripheral blood, and are predominantly located in secondary lymphoid organs. These cells primarily regulate the immune system by secreting cytokines such as interferon, tumor necrosis factor (TNF), and granulocyte macrophage colony-stimulating factor, among others [39]. Exocytosis of lytic granules, the release of pro-inflammatory cytokines, and cytotoxicity are all mediated by the interaction of signals obtained from two receptors with opposing effects, “germline-encoded inhibitory and activating receptors.” These receptors bind to corresponding homologous ligands on target cells, such as classical or non-classical major histocompatibility complex (MHC) class I, allowing NK cells to distinguish between transformed and normal cells [36, 40, 41]. The activating NK cell receptors that stimulate the destroying effects of these cells include the natural cytotoxicity receptor (NCR) family (NKp30, NKp44, and NKp46), the C-type lectin-like activating receptors (NKG2D, CD94/NKG2F, CD94/NKG2E, CD94/NKG2C, and CD161), activating killer immunoglobulin receptors (KIR2DS1, KIR2DS4 and KIR2DL4), FcγRc IIIA (CD16), and costimulatory receptor DNAM-1 (CD226) [42]. Killer cell immunoglobulin-like receptors (KIRs) and the heterodimeric C-type lectin receptor (NKG2A) are germline-encoded inhibitory receptors that engage with MHC molecules on normal cells and transmit inhibitory signals to NK cells, resulting in the development of NK cell tolerance to normal cells under healthy conditions and preventing NK cell-mediated lysis of normal cells [43, 44].

When healthy cells become infected with a virus or undergo transformation and malignancy, the expression of cell-surface molecules changes, preventing inhibitory reactions and activating NK cells, which then eliminate the transformed cells. Two theories explain how NK cells contribute to tumor immunosurveillance: missing self and induced self [45]. When NK cells recognize malignantly transformed cells with reduced expression of MHC class I molecules, they do not receive inhibitory signals from their inhibitory receptors, allowing these abnormal cells to be quickly lysed, according to the missing-self hypothesis [35, 46]. Induced self-mechanism displays increased expression of stress ligands on transformed cells due to elevated cellular stress factors and DNA damage, which can bind to NK cell-stimulating receptors such as NKG2D, resulting in NK cell activation and tumor cell destruction. As a result, the destruction of tumor cells by NK cells is mediated by a mixture of two pathways [47, 48]. Activated NK cells can mediate cytotoxicity against cancerous cells in a variety of ways. Via intrinsic cytotoxic action, formation of an immunologic synapse with transformed cells, and then exocytosis of cytotoxic granules containing perforin and granzymes, as well as the secretion of immune pro-inflammatory cytokines, activated NK cells may lyse tumor cells directly and acutely in a non-specific manner [49, 50]. Another pathway involves Fas ligand or TNF-related apoptosis-inducing ligand (TRAIL) “members of the tumor necrosis factor (TNF) family” interacting with tumor cells and inducing apoptosis [51, 52]. Target cells can also be eliminated by other pathways, such as antibody-dependent cell cytotoxicity (ADCC), which causes IgG-opsonized cells to be eliminated after binding to the CD16 receptor on NK cells, resulting in a robust immune response to tumors [50, 53]. NK cells also secrete cytokines including INF-gamma, which have pleiotropic effects and regulate antitumor responses in the tumor microenvironment by influencing the activity of activated T cells, macrophages, and dendritic cells [54].

Various NK cell-based adoptive immunotherapies have emerged as a result of the significant antitumor function of NK cells. Increase the activity of endogenous NK cells with cytokines, especially IL-2, is one of the primary strategies in tumor therapy. In vitro and In vivo, cytotoxicity and proliferation ability of NK cells enhance after treatment with exogenous cytokines such as Interleukins-2, -12, -15, -18, and -21. In patients with lung cancer, systemic administration of IL-2 was found to improve NK cell cytotoxicity. Unfortunately, some limitations, such as extreme cytokine toxicity and IL-2-based T lymphocyte regulator stimulation, restrict the strategy’s effectiveness and clinical use [55, 56]. Another strategy for enhancing NK cell activation and cytolytic properties has been the design of therapeutic monoclonal antibodies that bind to and suppress inhibitory receptors on NK cells [57, 58]. Adoptive cell therapy (ACT) has been developed in recent years to overcome the limitations of previous therapies. This approach involves isolating NK cells from autologous or allogeneic sources, expanding them in vitro, enhancing their activation and cytotoxicity by cytokine priming or co-culture of isolated NK cells with feeder cell lines, and then returning these modified NK cells to the patient’s body [59, 60]. Since self-HLA molecules on tumor cells suppress the cytotoxicity of autologous NK cells, NK cells from allogeneic origins seem to be a potential alternative therapeutic target for adoptive immunotherapy [61]. In recent years, emerging research has shown that allogeneic NK cells can be used to treat a wide variety of hematological malignancies and solid tumors [60]. Furthermore, it has previously been explored whether genetically modified NK cells, such as those engineered to express tumor-targeting CARs, could improve their effectiveness in destroying tumor cells [2].

## CAR-NK cell generation

### Source of NK cells for the manufacture of CAR-NK cells

**Fig. 2:**
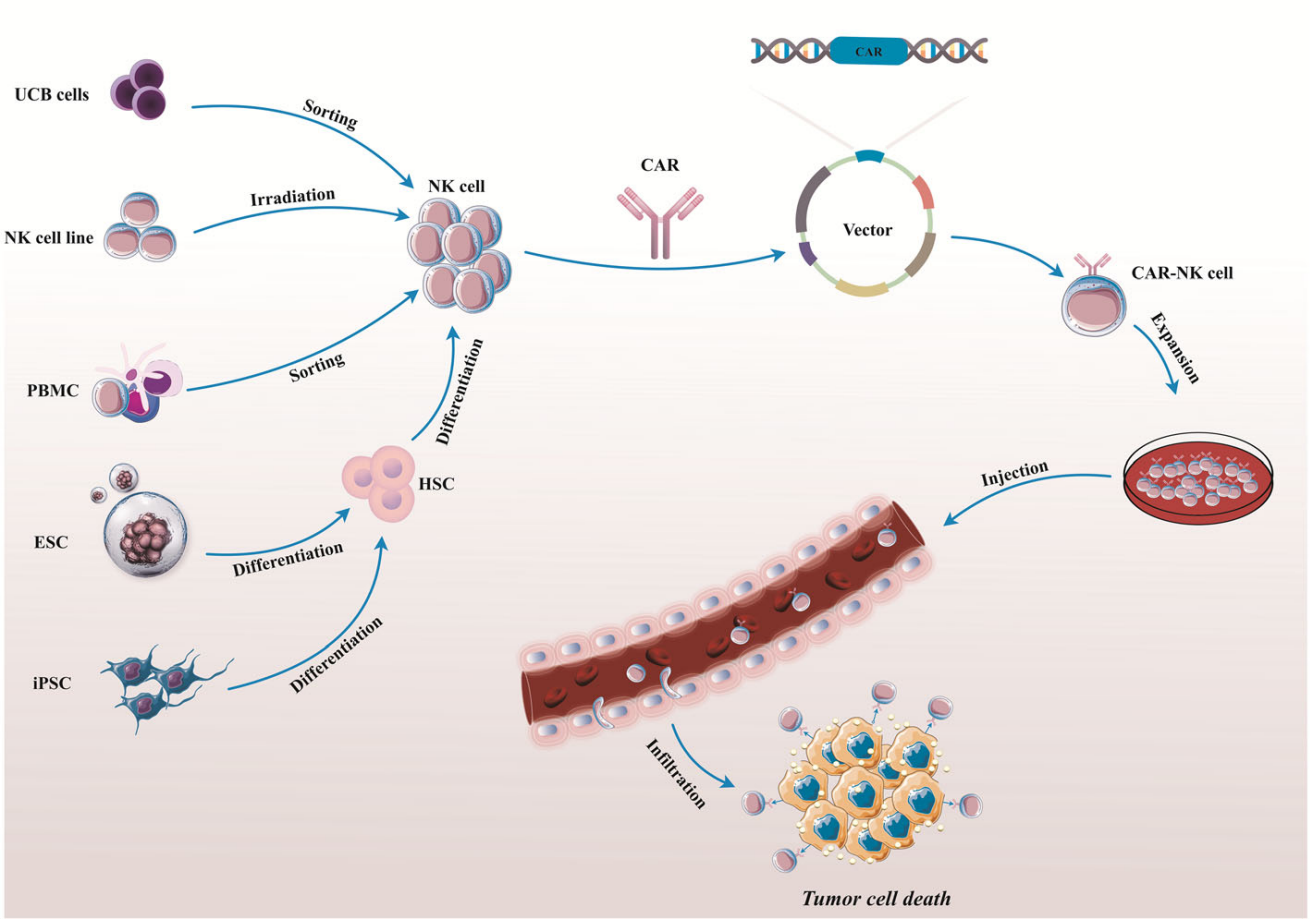
The CAR-NK cell production process. Procured or established NK cells derived from multiple sources, such as PBMC, UCB, HSCs, ESCs, and iPSC can be modified with CAR-expressing vectors, and then be cultured in NK cell-specific expansion media with particular cytokines to finally generate CAR-redirected NK cells. Umbilical cord blood (UCB), peripheral blood mononuclear cell (PBMC), induced pluripotent stem cell (iPSCs), embryonic stem cell (ESC), hematopoietic stem cell (HSC), chimeric antigen receptor-natural killer (CAR-NK) cells

Functional NK cells for adoptive cell-based immunotherapy can be obtained from a variety of cell sources, and then be modified to establish CAR-redirected NK cells (Fig. 2) [62]. Allogeneic NK cells can be generated from a variety of sources, including peripheral blood (PB) [63], easily accessible NK cell lines [62, 64], and, more recently, stem cells such as umbilical cord blood (UCB) [65], human embryonic stem cells (hESCs) [66], and induced pluripotent stem cells (iPSCs) [67, 68]. Each source has its own set of advantages and disadvantages. Peripheral blood NK cells collected from a donor by lymphocyte apheresis have favorable features of relief and safety, as well as expressing a huge number of activating receptors such as CD16, NKG2D, and the NCRs (NKp44 and NKp46) equipped with KIRs after activation, which plays an important role in NK cell licensing and has a powerful destructive ability against abnormal cells [69, 70]. However, collecting NK cells from peripheral blood (PB) is time-consuming and expensive; on the other hand, peripheral blood mononuclear cells (PBMCs) contain a low percentage of NK cells under normal conditions [71]. As a result, techniques for extending and activating NK cells have appeared. Among these, stimulatory cytokine exposure or co-culture of NK cells with feeder cells are existing methods for obtaining a large number of NK cells and activation [37, 72]. Clonal NK cell lines, such as NK-92, NK-YS, NKL, NKG, KHYG-1, and others, are also essential cellular origins for allogeneic CAR-NK cell therapy [73]. NK-92 cells are well-defined and have effective anticancer properties [74]. NK-92 cells secrete more perforin, granzyme, and several cytotoxic cytokines than PB NK cells. Furthermore, unlike PB NK cells, these cells can be quickly multiplied without the use of feeder cells or under difficult and complicated conditions, and they generate a more homogeneous cell population [75, 76]. However, NK-92 cells lack the expression of several common activating receptors, such as NKp44 and NKp46, and the lack of inhibitory KIRs may limit their potent cytotoxic effects [77]. Consequently, they lack CD16 (FCRIII) expression and hence are unable to mediate antibody-dependent cell cytotoxicity (ADCC) [78]. Moreover, to mitigate the likelihood of perpetual allogeneic tumor engraftment and tumorigenicity, the NK-92 cell line must be irradiated before infusion, which inhibits their durability and expansion in the host [78]. Another potential source of allogeneic NK cells and “off-the-shelf” products for immunotherapy treatment is umbilical cord blood (UCB). UCB NK cells are naturally immature, with lower levels of activating receptors including NKp46, NKG2C, IL-2R, DNAM-1, CD57, adhesion molecules as CD2, CD11a, CD18, CD62L, and CD16, and a higher level of the inhibitory receptor NKG2A [79, 80]. So, following ex vivo amplification and activation of these cells, which require exposure to combinations of stimulatory cytokines or donors, they mature and express a broad range of activating and inhibitory receptors [81]. The significant advantages of UCB NK cells include their relative speed and convenience in the collection, significant proliferative ability, and reduced probability of GvHD [82, 83]. CD34-positive cells, such as iPSCs, have recently been identified as an additional source of NK cell production. Under standardized culture conditions, iPSC-NK cells have a significant amplification potential to generate a large amount of homogeneous NK cells with potent cytotoxic activity [84–86]. These cells have a normal rate of killer activating receptors such as NKG2D, NKp46, Fas, and TRAIL expression, but a lower rate of KIR expression than peripheral blood NK cells [87]. Moreover, iPSC-NK cells are surprisingly simple to genetically modify with altered characteristics, making them a promising technique in immunotherapy [78].

### Structure of CAR

**Fig. 3:**
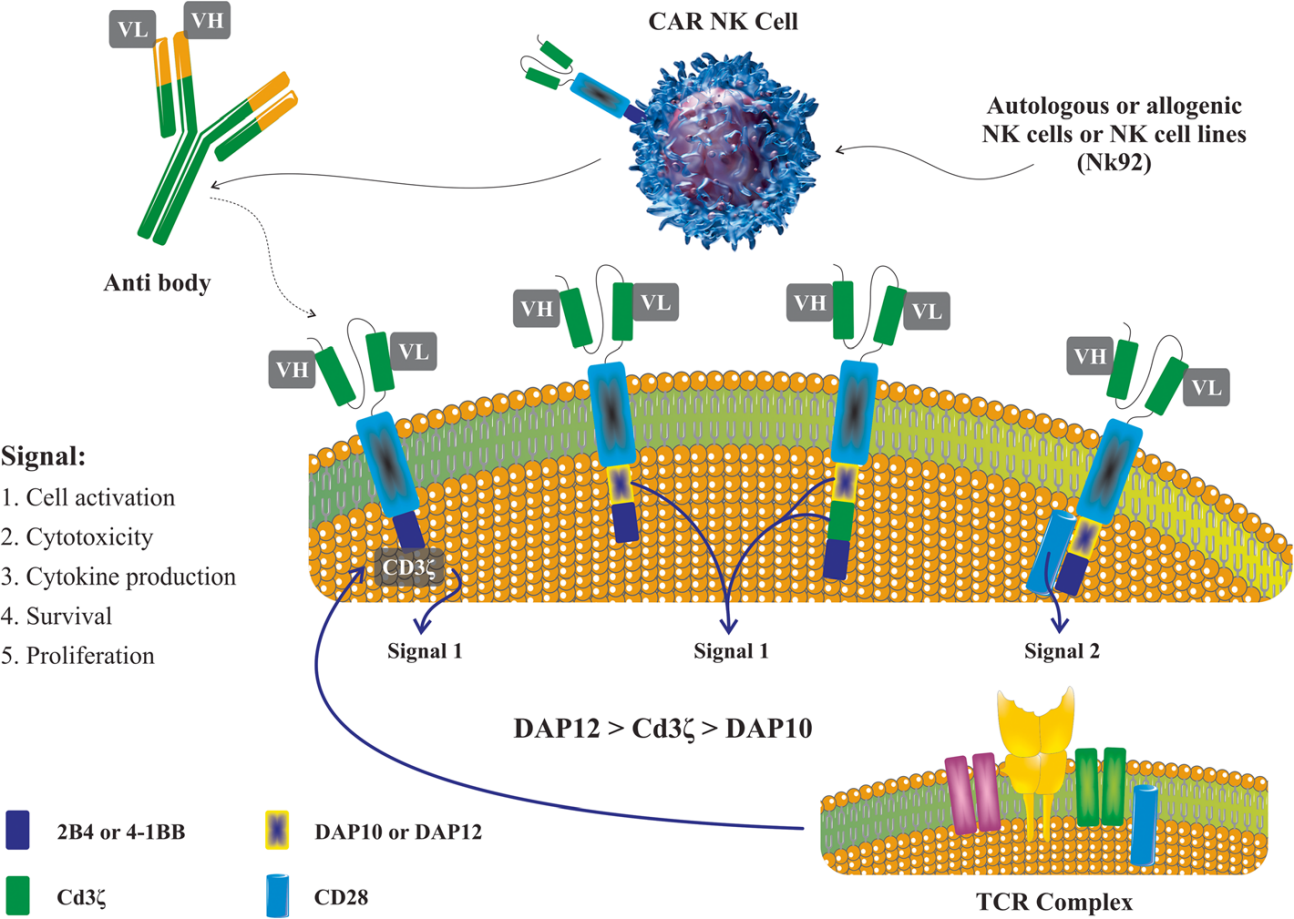
The common CAR constructions utilized in CAR-NK cell generation. CAR molecules on NK cells include three chief fragments, comprising an antigen detection domain (ScFv or NKG2D), and transmembrane domain concomitant with the signaling domain. First-generation CARs only include CD3ζ or DAP12 as the signaling domain, and CD3ζ seems to be a more efficient signaling domain than DAP10, while DAP12 can stimulate NK cell functions more powerful than CD3ζ. Besides, second-generation CARs express a second signaling domain, including CD28 or 4-1BB in association with CD3ζ. Finally, third-generation CARs include two costimulatory signaling domains. Importantly, respecting the mechanism by which NKG2D induces NK cells, an exclusive CAR construct including NKG2D as the ectodomain that connects DAP10 and CD3ζ as chief signaling domains has been progressed. Chimeric antigen receptor-natural killer (CAR-NK) cells, natural killer group 2D (NKG2D)

The design of an appropriate CAR construct for the genetic modification of NK cells, similar to CAR-T cells, helps to increase the cytotoxic potential of these cells for specific antigens in targeted cells. CAR-NK is composed of a main CAR-T construct, an extracellular antigen identification domain (typically a single-chain variable antibody fragment (scFv)), a transmembrane domain, and an intracellular signaling domain [88]. The CAR’s evolution is currently summarized in four generations. In the first generation of CARs, both CAR-T and CAR-NK typically use CD3ζ as a single activation intracellular signaling region [89]. Following that, as second- and third-generation CARs, additional cost-stimulatory signaling regions, such as CD28 or CD137 (4-1BB), were introduced to improve the efficiency of NK cells (Fig. 3) [90]. While 4-1BB-composing CARs are used in both T and NK cells, the role of CD28-composing CARs in NK cells has received less attention [91]. As a result, subsequent studies have designed CAR structures based on NK cell characteristics and the use of more specific costimulatory regions such as DAP10, DAP12, or 2B4 (Fig. 3) [92–94]. The main intracellular domains for signal transmission of NK stimulatory receptors are DNAX-activation protein 12 (DAP12) and DAP10 (Fig. 3). DAP12 is required for the activating receptors NKG2C, NKp44, and KIR, while DAP10 is necessary for NKG2D costimulatory signaling [95]. Anti-CD19 CARs containing both signaling domains DAP10 and CD3ζ showed a more efficient cytotoxicity response than either domain alone in the CAR-NK structure [96]. Moreover, DAP12-incorporated CAR constructs show more apparent promise in a range of primary NK cells or NK92 cell lines than NK cells with CD3ζ-containing CAR constructs [97]. Besides that, using the 2B4, a prominent NK-specific costimulatory domain, to generate anti-CD5 CAR-NK cells resulted in rapid proliferation, specific cytotoxicity, and stronger anti-malignant efficacy against T cell malignancies in vitro and T-ALL xenograft mice [98]. The function of CAR-NK cells in tumor inhibition has been stated not only by the ability of the CAR construct to detect tumor-specific antigens but also by their natural receptors, which are not antigen-specific, for the transmission of stimulatory signals within NK cells and the activation of various killing mechanisms have a decisive role to play in explaining the behavior of NK cells in the tumor microenvironment [99]. Recently, the fourth generation of CARs carrying a transgenic “payload”' such as IL-2 or IL-15 has been engineered to improve CAR-NK cell proliferation, longevity, and cytotoxicity against antigen-negative tumor cells. To date, the data published affirm the specific features of these latest CAR constructs as a possible immunotherapeutic approach to modulating the tumor microenvironment [100–102].

### CAR transduction into NK cells

To progress in the field of CAR-NK cell immunotherapy, an appropriate method for transferring genetic material into NK cells must be used. So far, two kinds of vectors based on viral or non-viral systems have been extensively used in CAR gene transduction. Vectors based on retroviruses or lentiviruses are more commonly used in genetic modification strategies. During retroviral RNA genome integration, viral RNAs are reverse-transcribed into double-stranded cDNA, which is then semi-randomly inserted into the host genome with the assistance of viral integrase [78]. Vectors developed by these viral pathogens have shown considerable advantages, which are determined by their features and making them comparatively convenient to inject into host cells. Typically, an adequate amount of retroviral generate is used for efficacious gene transfer in the manufacturing process, and vectors are stably integrated into the host DNA. Retroviral vectors can hold transgenes of acceptable sizes (7–8 kb) [103], and their transduction efficiency for primary NK cells from peripheral blood is high (43–93%) [96]. However, this approach has several potential drawbacks, including vector instability and the failure to infect non-dividing cells. Besides that, retroviral vectors integrate into the host genome at random, raising the possibility of insertional mutagenesis and other harmful implications [4, 104]. In comparison to retroviral vectors, lentivirus-based vectors infect both dividing and non-dividing cells and allow for the introduction of larger transgenes up to 10 kb in size, as well as a lower likelihood of insertion mutagenesis, but have a lower capacity to inject CARs into primary NK cells, which needs an overhaul [54, 103]. The transduction efficiency of lentivirus-based vectors, on the other hand, is reasonable for NK cells derived from cord blood [105]. Resistance of NK cells to viral-based gene delivery and lower transfecting efficiency of NK cells compared to T cells stem from NK cells’ natural capacity, as an essential cytotoxic member of the innate immune system, to defend against viral infection guided by pattern recognition receptor signaling [106, 107]. As a result, suppressing innate immune responses to viral infection can improve lentivirus transfection of NK cells and establish viral vectors as an effective and safe strategy for NK cell gene modification. According to research, the use of BX-795, an inhibitor of 3-phosphoinositide-dependent kinase 1 (PDK1), negatively controls signaling pathways of RIG-I, MDA-5, and TLR3 that are involved in the antiviral response, and thus enhances the transduction efficiency of lentiviral vectors by 3.8-fold on average [108].

When compared to viral approaches to gene alteration, non-viral-based gene modifications are more stable, easier to synthesize, can transduce larger genetic payloads (> 100 kb), and are cost-effective for therapeutic use. As a result, non-viral vectors have emerged as a more effective alternative to viral vector-based CAR integration techniques [109–112]. Transposon-based mechanisms, such as the Sleeping Beauty (SB) transposon vector, have been used successfully to deliver transgene into the host genome as an alternative to viral vectors. Transposon vectors are made up of a transposon with the CAR gene sequence flanked by inverted terminal repeats (ITRs) and a transposase (e.g., SB100X) that binds to ITRs and mediates random transposon entry into the host genome [4, 113, 114]. SB transposon systems have been developed to produce safe and effective CAR-T cells for preclinical and clinical research [115, 116]. However, the feasibility of using an SB transposon system to deliver DNA plasmids as CAR sequence carriers into NK cells is debatable and unknown [4]. Moreover, genetically modifying NK cells through electroporation of DNA or mRNA plasmids is a promising non-integrating strategy. While electroporation of DNA plasmids has low efficiency, electroporation of mRNA is an accurate and cost-effective tactic in clinical applications [105, 117]. Xiao et al. observed that > 95% of primary NK cells expressed NKG2D RNA CAR and that NK cell viability after mRNA electroporation was greater than 90% before infusion [118]. Notably, mRNA electroporation efficiencies of 80–90% were observed in both ex vivo-expanded NK cells and primary resting (non-cytokine stimulated) human NK cells [119]. Nonetheless, owing to constant permeability in the membrane and the failure of cell homeostasis, electrical pulses in the electroporation tactic will often result in the loss of a variety of cells [120, 121]. Besides that, a major drawback of most non-viral gene vectors, such as electroporation, is the transgene’s unstable and transient expression, which could necessitate additional doses of adoptively transferred immune cells to achieve efficient therapeutic function; nevertheless, additional doses may be problematic and have their conflicting consequences [122–124].

To conclude, since transferring genetic material into NK cells and scaling up these transformed cell products is challenging, selecting a more appropriate and efficient technique that adheres to existing good manufacturing practices (cGMP) standards is an essential step toward successful clinical trials.

## Advantages of CAR-NK cell immunotherapy

Despite the recent success of CAR-T cell immunotherapy in the treatment of hematological tumors [125, 126], FDA approval is a major step in the evolution of genetically engineered cell-based tumor therapies. However, there are also flaws in the extensive therapeutic application of CAR-T cell therapy. On the other hand, the distinct biological properties of NK cells, as well as some benefits of NK cell therapy over CAR-T cell therapy, have sparked significant interest in the application of CAR-NK cells and their development for cancer immunotherapy [99, 127, 128].

First, preclinical and some phase I/II clinical trials have shown that allogeneic NK cell infusions decrease the risk of GVHD and serious harmful impacts. As a result, NK cells are acceptable CAR drivers that are not restricted to autologous cells [60, 129–133]. Second, mature NK cells have a comparatively shorter lifespan in the bloodstream, reducing the possibility of profound and long-term cell deficiency due to cellular memory responses and on-target/off-tumor effects such as B cell deficiency (in the case of CD19-targeting CAR-T cells) [133, 134]. Third, the cytokines secreted by activated NK cells were assumed to be safer and typically consisted of IFN- and granulocyte macrophage colony-stimulating factor (GMCSF), which eliminated the risk of cytokine storm and extreme neurotoxicity caused by pro-inflammatory cytokines such as TNF-, IL-1, and IL-6 in CAR-T cell therapy [127, 135]. Fourth, CAR-NK cells maintain their natural intrinsic ability to recognize stress-evoked ligands presented on tumor cells through their native activating receptors, such as natural cytotoxicity receptors (NKp46, NKp44, and NKp30), NKG2D, and DNAM-1, unlike CAR-T cells, which eliminate cancer target cells only by identifying the tumor-associated antigen through scFv in CAR-related mechanisms. These stress-induced ligands are found on tumor cells during an initial interaction with immune cells or during long-term treatment. As a result, CAR-expressing NK cells can effectively eradicate heterogeneous malignancy in which some malignant cells lack CAR-targeted specific antigen via both CAR-dependent and NK cell receptor-dependent pathways [9, 136, 137]. Fifth, the minimal risk for alloreactivity and GVHD potentially permits allogenic CAR-NK cells to be procured from various sources, including PB, UCB, hESCs, iPCSs, and even NK-92 cell lines. For instance, homogeneous NK92 cells provide an “off-the-shelf” CAR-engineered manufacture for wider clinical applications, while the CAR-NK cells generated from genetically modified human iPSCs can be produced a homogeneous population and have presented strong anticancer capacity and proliferative capability in preclinical studies [4, 138].

## The challenges faced by CAR-NK cells

Despite the potential advantages of NK cells, there are several challenges to using CAR-NK cells in clinical trials. The first reluctance stems from the structure of the current CARs used in NK cells. The position of the epitopes binding the current CAR, as well as their distance from the CAR-NK cell’s surface reduces these cells’ ability to bind antigens and stimulate CAR-NK cells. To eliminate melanoma, Li et al. generated an optimized construct of CARs for NK cell activation and cytotoxicity by concentrating on intracellular excitation adapter molecules [76, 139].

The second issue comes from the lack of an efficient gene transfer approach in NK cells. Although viral transfection is a promising method for T cells, it results in low levels of transgene expression in NK cells and adversely impacts their survival, as stated in previous sections. Non-viral vectors minimize these disadvantages and are regarded as desirable and safe alternatives. However, it is unclear whether all of these non-viral vectors are appropriate for CAR-NK constructs [38, 62, 140].

The third concern with using NK cells for immunotherapy is that they are highly vulnerable to freezing and thawing, and their activation and cytotoxicity are greatly reduced after thawing. Many studies have shown that incubating frozen NK cells with cytokines such as IL-2 may restore their function. As a result, strategies for desirable cryopreservation and optimum restoration of activity of frozen CAR-NK cells for adoptive therapy must be investigated [141, 142].

Fourth, the reality that infused cells do not persist in the absence of cytokine assistance is perhaps the most significant challenge of appropriate CAR therapies. While these features of NK cells can be beneficial, they may also restrict the effectiveness of NK cell immunotherapy. For infused NK cells to survive and proliferate in vivo, exogenous cytokines must be administered sequentially [143]. Exogenous cytokines, on the other hand, have undesirable side effects and can promote other immune subsets such as regulatory T cells, which may be immunosuppressive to NK cells [144, 145]. A unique approach is to incorporate cytokine transgenes into CAR-engineered NK cells, which continuously supply cytokine support [146, 147]. CAR.19/IL-15-transduced CB NK cells generated intense interleukin (IL)-15, which assisted their extended survival and enhanced proliferation in vivo. Furthermore, CAR.19/IL15+ CB NK cells outperformed CAR.19-transduced NK cells missing IL-15 in terms of antitumor function and survival in the elimination of B cell malignancies [148].

## Preclinical studies of CAR-NK cell in hematological malignancies

Even though CAR-T cell immunotherapy has reported promising results in the treatment of patients with hematological malignancies, treatment-related toxicity and side effects remain serious barriers. Natural killer (NK) cells, as an effective effector cell in innate immunity, have a great deal of antitumor capacity and have more promising prospects in the immunotherapy of hematologic malignancies. The purpose of creating CAR-NK cells is to use a novel strategy for activating NK cells and improving their antitumor properties by genetic modification to eventually produce “off-the-shelf” antitumor immunotherapeutic products [149]. Lymphomas and leukemias are a large group of hematological malignancies characterized by clonal growth and dysfunction of lymphoid and myeloid cells at different stages of maturation and commitment, with varying clinical outcomes and prognoses [150, 151]. A large number of preclinical trials have revealed that CAR-NK can effectively treat lymphomas and leukemias by targeting antigens such as CD19, CD20, CD7, and CD5 (Tables 1 and 2) [148, 158, 163, 174].

**Table 1:** Overview of in vitro studies based on CAR-NK cell therapy for hematological malignancies

*Condition*	*Target*	*Main outcomes*	*Ref*
B cell acute lymphoblastic leukemia (ALL) and T cell ALL	NKG2D	Secretion of IFN-γ, GMCSF, IL-13, MIP-1a, MIP-1b, CCL5, and TNF-ɑ, massive release of cytotoxic granules and efficient cytotoxic effects against T cell ALL (CEM-C7, MOLT-4, Jurkat) and B cell ALL (REH, OP-1) by NKG2D-DAP10-CD3z-expressing NK cells	[152]
B cell malignancies	CD19	The efficient killing of CD19-expressing cell lines and primary leukemia cells by iC9/CAR.19/IL-15-transduced cord blood (CB)-NK cells	[148]
B cell ALL and B cell chronic lymphocytic leukemia (CLL)	CD19	Higher anticancer activity of peripheral blood (PB)- CAR-NK cells compared with CB CAR-NK cells at killing CD19+ K562, Nalm-6 target cells, and ALL and CLL cells	[153]
B cell leukemia and lymphoma	CD19	Exposure of established cancer cell lines and primary pre-B-ALL blasts with NK-92/63.z and NK-92/63.28.z cells led to cell killing and cytokine production	[64]
B cell precursor acute lymphoblastic leukemia	CD19	Higher antileukemic activity toward CD19+ cell lines and primary blasts obtained from patients with B cell precursor ALL with CAR-CD19-PB NK cells	[154]
B cell acute lymphoblastic leukemia (BLL)	CD19	Specific cell killing activity against CD19-expressing Raji Burkitt’s lymphoma and primary B-ALL blasts by CD19-CAR-NK cells	[155]
Chronic lymphocytic leukemia (CLL)	CD19	Significant cytolytic function toward previously resistant CD19 positive cell lines and primary CLL cells by CD19-CAR-NK-92 cells	[156]
NK-resistant B cell lymphoma malignancies	CD19	Displaying significantly increased IFN-γ production, degranulation, and specific killing against NK-resistant lymphoma lines and primary targets by CD19-CAR-NK cells	[157]
Lymphoma and leukemia	CD20	Effective eliminating NK cell-resistant primary CLL by CD20-CAR-NK-92 cells	[158]
B cell non-Hodgkin’s lymphomas (NHL)	CD20	Improved cytotoxicity against rituximab-opsonized Raji and MAVER-1 CD20+ cell lines by NK-92MI cells expressing CD16-BB-ζ or CD64- BB-ζ receptors	[159]
CD20+ B- non-Hodgkin’s lymphomas (NHL)	CD20	Marked cytotoxicity against CD20+ Ramos, Daudi, Raji, and two rituximab-resistant cell lines (Raji-2R and Raji-4RH) by CD20-CAR PB NK cells	[160]
Burkitt Lymphoma	CD20	The combined treatment with romidepsin and CD20-CAR-PB NK cells significantly induced cell death in Burkitt Lymphoma cell lines such as Raji, Raji-2R, and Raji-4RH cells	[161]
pre-B cell acute lymphoblastic leukemia (B-ALL)	FLT3	Exposure of FLT3-positive B-ALL cell lines and primary blasts with CAR NK-92 cells resulted in NK-cell degranulation and selective cytotoxicity	[162]
T cell leukemia and lymphoma	CD5	Eliminating both CD5+ tumor cell lines and CD5+ primary tumor cells in vitro by CD5-CAR-NK-92 cells	[163]
T cell malignancies	CD5	Notable cytotoxicity against the CD5-positive Jurkat and MOLT-4 leukemia cells by CD5-CAR-expressing NK-92 cells	[164]
T cell malignancies	CD5	CD5-CAR-NK cells with costimulators 2B4 displayed greater anti-CD5+ cytotoxicity than CD5-CAR-NK with costimulators 4-1BB against CD5+ malignant cell lines, and primary CD5+ malignant cells through upregulation of activation markers and cytotoxic granule release	[98]
T cell non-Hodgkin’s lymphomas (NHLs)	CD4	Robustly eliminating diverse CD4+ human T cell leukemia and lymphoma cell lines (KARPAS-299, CCRF-CEM, and HL60) and primary CD4+ T cell malignancies by CD4-CAR-NK-92 cells	[165]
Multiple myeloma	CS1	Improved cytotoxicity against CS1+ MM cell lines and IFN-γ production with CS1-CAR-NK-92 and CS1-CAR-NK cells	[166]
Multiple myeloma	CD138	Significant cytotoxicity and secretion of granzyme B, IFN-γ, and proportion of CD107a expression in CD138-CAR-NK-92MI cells in response to CD138-positive human MM cell lines (RPMI8226, U266, and NCI-H929)	[167]
Multiple myeloma	NKG2D	Primary NK cells from MM patients transduced with NKG2D-CARs showed considerably cytotoxic activity against the majority of MM cell lines	[168]
Acute myeloid leukemia (AML)	CD123	Recognition of CD123 + AML cell line KG1a and primary AML blasts and enhanced secretion of TNF-ɑ, IFN-γ and granzyme A and B along with showing significant cytotoxicity against listed cell lines	[169]
Acute myeloid leukemia (AML)	CD123	More prominent cytotoxic activity and secreting higher granzyme A and IL-17A levels against the CD123+ AML cell line KG-1a and primary human AML cells by CAR-NK-92 than CAR-PB NK	[170]
Acute myeloid leukemia (AML)	CD123	Cytolytic functions in association with perforin and granzyme production against CD123 expressing AML cell lines upon exposure with CD123 CAR-NK-92	[171]
Acute myeloid leukemia (AML)	CD123	CD123-CAR-CB NK cells showed more antileukemic activity and higher secretion of TNF-ɑ, IFN-γ against CD123+ AML cell lines (THP-1 and MOLM-14)	[172]
Acute myeloid leukemia (AML)	CD4	Elimination of CD4+ AML cell lines THP-1, U937, and MOLM-13 and CD4+ human primary AML cells by CD4-CAR-PB NK cells	[173]

**Table 2:** Overview of in vivo studies based on CAR-NK cell therapy for hematological malignancies

*Condition*	*Target*	*Main results*	*Ref*
B cell malignancies	CD19	Prolonged survival in a xenograft Raji lymphoma murine model upon injection iC9/CAR.19/IL-15-transduced CB NK cells which produce IL-15 to improve their function	[148]
B cell precursor acute lymphoblastic leukemia	CD19	Potent antileukemia activity of human lymphoma in (NSG) xenograft mice model by CAR-CD19-PB NK cells	[154]
B cell leukemia and lymphoma	CD19	Abrogation of disease progression with selective cytotoxicity against Raji B cell lymphoma xenograft NSG mice model upon injection of NK-92/63.z cells	[64]
B cell acute lymphoblastic leukemia (BLL)	CD19	Complete molecular remission and prolonged survival in B cell lymphoma xenograft (NSG) mice model by CD19-CAR-NK cells	[155]
Lymphoma and leukemia	CD19 & CD20	Eradication of TMD-5 (CD19 + CD20+) cells by Intrafemoral injection of CD19-CAR NK-92 and eliminating BCR-ABL1+ SUP-B15 (CD19 + CD20−) cells by intravenous injection of CD19-CAR NK-92 in xenotransplant mouse models Effective suppressing local tumor development in Daudi lymphoma xenograft mice model by CD20-CAR NK-92 than CD19-CAR NK-92	[158]
B cell non-Hodgkin’s lymphomas (NHL)	CD20	Inhibiting MAVER-1 tumor cell growth in xenograft NCG mice model with NK- 92MI cells expressing receptor of CD16-BB-ζ	[159]
CD20+ B cell non-Hodgkin’s lymphomas (NHL)	CD20	Reducing tumor size and extended survival in Raji-Luc and Raji-2R-Luc xenograft NSG mice model upon injection of CD20-CAR-PB NK cells	[160]
Burkitt Lymphoma	CD20	The combination of romidepsin and CD20-CAR-PB NK cells reduced tumor burden and enhanced survival in humanized BL in xenograft NSG mice models	[161]
Pre-B cell acute lymphoblastic leukemia (B-ALL)	FLT3	Abrogated disease progression, high antileukemic activity, and enhancing safety by NK-92 cells co-expressing the FLT3-specific CAR and iCasp9 in a B-ALL xenograft model in NSG mice	[162]
T cell acute lymphoblastic leukemia	CD5	Abrogated disease progression and improved survival with CD5-CAR-NK-92 cells in xenograft mouse models of CD5+ T-ALL	[163]
T cell malignancies	CD5	A significant decrease in tumor burden was observed with CD5-CAR-expressing NK-92 cells in a T cell leukemia xenograft mouse model	[164]
T cell malignancies	CD5	CD5-CAR-NK cells with costimulators 2B4 showed superior cytotoxic ability against T-ALL in mouse xenograft models and prolonged the survival of T-ALL xenograft mice than CD5-CAR-NK with costimulators 4-1BB	[98]
T cell non-Hodgkin’s lymphomas (NHLs)	CD4	CD4-CAR-NK-92 cells significantly reduced tumor burden and prolonged survival in KARPAS-299-injected NSG mice	[165]
Multiple myeloma	CS1	Suppressing the growth of human IM9 MM cells and also significantly prolonged survival in an aggressive orthotopic MM xenograft mouse model upon injection of CS1-CAR-NK-92 cells	[166]
Multiple myeloma	CD138	Marked antitumor activity toward CD138+ MM cells in the xenograft SCID mouse model by CD138-CAR-NK-92MI cells	[167]
Acute myeloid leukemia (AML)	CD123	Significantly reduced disease burden in NSG mice xenografted with luciferase-expressing THP-1 cells upon injection of CD123-CAR-NK-92	[171]
Acute myeloid leukemia (AML)	CD4	Antileukemic effects in a systemic AML murine model with CD4-CAR-PB NK cells	[173]

### CAR-NK cells in leukemia

Extensive studies on the application of CAR-NK cells in the treatment of various forms of Leukemia have just been initiated. By lentiviral gene transfer to CD19-specific CARs that attacked CD19-positive cells and carried a CD3 signaling endodomain either alone or with a costimulatory domain, Oelser et al. transduced a heterogeneous effector cell population defined as cytokine-induced killer (CIK) cells (CD28 or CD137). In vitro, these targeted CIK cells exhibited selective cytotoxicity against cancer cell lines and Primary Pre-B- acute lymphoblastic leukemia (ALL) blasts [155].. Other research groups later confirmed that CAR-engineered NK92 cells had an antileukemic function and could overcome B cell acute and chronic leukemia resistance to parental NK cells. The NK-92 cell lines can be transfected with high transduction efficiency and minimal effect on cell viability using electroporation of CD19-CAR mRNA [156]. In vitro, Boissel et al. reported that NK-92 cells expressing anti-CD19 CAR had effective cytolytic activity against previously resistant CD19-positive B-ALL cell lines and primary chronic lymphocytic leukemia (CLL) [158]. NK-92 cells were also transduced with a lentiviral gene that encoded an FMS-like tyrosine kinase 3 (FLT3)-specific CAR with a composite CD28-CD3 signaling domain and an inducible caspase-9 (iCasp9) suicide gene to improve therapeutic utility and safety. FLT3-specific CAR-NK-92 cells with FLT3 expression and CAR activation were used to target B-ALL cell lines and primary blasts that had altered or lost CD19 expression and were resistant to parental NK-92 in this study. In addition, FLT3-specific CAR-NK-92 cells expressing the iCasp9 suicide gene were readily inactivated in the event of serious side effects [162]. A group of researchers looked into the ability of CAR-engineered NK-92 cells and parental NK-92 cells expressing FcRIII plus anti-CD20 monoclonal antibodies (MAbs) like rituximab to kill NK cell-resistant B-lymphoid leukemia. In vitro observations indicate that CD20-specific CAR-NK-92 cells more effectively eliminated NK cell-resistant primary CLL cells than NK-92 and ADCC arising from it with the participation of anti-CD20 MAbs. They employed two ALL cell lines, BCR-ABL1+ SUP-B15 (CD19 + CD20−) and TMD-5 (CD19 + CD20+), to create two xenotransplant mouse models of residual leukemia for in vivo studies. SUP-B15 cells were killed by intravenous injection of CD19-specific CAR-NK-92, but TMD-5 cells resisted the immune attack. Interfemoral injection of CD19-specific CAR-NK-92, on the other hand, was a method of removing TMD-5 cells from the bone marrow environment. As a consequence, it seems that the process of administration affects the treatment outcome, owing to permeability and local concentration [158]. UCB is a noteworthy and allogeneic source of NK cells in addition to NK-92. CAR-NK was transduced from CB with a structure that included coding segments for CAR for CD19, interleukin (IL)-15 (to increase cell proliferation and persistence), and an inducible caspase-9 (iC9)-based suicide gene, according to Liu and his colleagues. The CAR-modified NK’s antitumor activity and survival were significantly improved by the synthesis of IL-15, compared to normal CAR-NK, and the expression of iC9 improved the cells’ safety [101].

CAR-NK has also shown promise as a successful curative therapy for aggressive T cell malignancies. CARs targeting CD5 or CD3 that are delivered into the NK-92 cell line have been found to have efficient cytotoxic activity against primary peripheral T cell lymphoma cells and T cell leukemia cell lines [163, 175]. Among these, CD5 is one of the significant characteristic markers and potent target for T cell malignancy, and NK cells, which are CD5-negative, may be efficacious for the immunotherapy of T cell malignancy. Chen et al. used lentiviral gene transfer to transform human NK cell line NK-92 into anti-CD5 CAR (CD5CAR), which could target several T cell leukemia and lymphoma cell lines as well as primary tumor cells and contained the intracellular signaling domain CD3 zeta either with two costimulatory domains (CD28 and 4-1BB). They demonstrated that CD5CAR-NK-92 cells specifically target CD5+ tumor cell lines and CD5+ primary tumor cells in vitro and exert consistent, potentially cytotoxic effects. In addition, an in vivo study of T-ALL xenograft mouse models revealed effective tumor progression restriction and control, supporting the efficacy of CAR-NK in T cell malignancies [163]. Besides, in another research, two anti-CD5 CAR plasmids with distinct costimulatory domains were designed; one utilized T cell-related activating receptor-4-1BB (BB.z) and the other utilized NK-cell-related activating receptor-2B4 (2B4.z). By the upregulation of activation markers and cytotoxic granule release, 2B4.z-NK cells demonstrated rapid proliferation and superior antitumor potency in both malignant CD5+ cell lines and primary CD5+ malignant cells in vitro. Moreover, employing mouse xenograft models to investigate the cytotoxic effects, it was found that CAR-2B4-NK cells were more effective than CAR-4-1BB-NK cells [98].

Acute myeloid leukemia (AML) is a heterogeneous aggressive disorder characterized by high proliferation, clonal expansion, and faulty and irregular cell differentiation of hematopoietic progenitors/precursors in the bone marrow and peripheral blood. The lack of satisfactory findings in AML immunotherapy is due in part to the shared expression of phenotypic markers with normal HSCs and in part to AML gene expression heterogeneity [176]. CAR-NK cells may have the ability to overcome treatment challenges and improve the results of AML immunotherapy because AML blasts contain ligands that can be detected by activating receptors in NK cells [177]. According to research, CD123 CAR-NK cells could be a promising immunotherapy component for treating all acute leukemias with high CD123 expression, including AML and B-ALL. In vitro, the NK cell line NK92 and PB NK cells engineered with self-inactivating (SIN) alpha-retroviral vectors to express the third-generation anti-CD123 CAR constructs showed strong antitumor activity against the CD123+ AML cell line KG-1a and primary human leukemia cells, according to the findings. While CD123-CAR-NK-92 cells and CD123-CAR-NK cells both improved antileukemic function as compared to unmodified or EGFP-modified NK cells, CD123-CAR.NK-92 cells showed significantly greater activity against AML cells [170]. In another analysis, peripheral blood NK cells were expanded with irradiated (25 Gy) autologous feeder cells in the presence of IL-21, and then engineered with alpha-retroviral self-inactivating (SIN) vectors to express anti-CD123 CAR with CD28 and 4-1BB (CD137) costimulatory signaling endodomains, as well as the CD3f signaling domain. In vitro, Klob et al. found that these modified cells had improved antitumor function, with enhanced degranulation and higher secretion of tumor necrosis factor-alpha, interferon-gamma, and granzyme A and B [169]. CB NK cells were reprogrammed by retroviral transduction to express anti-CD123 CARs comprising coding parts for interleukin (IL)-15 (to support NK cellular survival and proliferation) and inducible caspase-9-based suicide gene (iC9), in other preclinical models. When compared to non-transduced NK cells, these modified NK cells displayed more antileukemic activity and higher secretion of IFN-gamma and TNF-α in vitro against CD123 + ve AML cell lines (THP-1 and MOLM-14) [171]. CD4 is another target expressed in certain AML subsets. The CD4CAR lentiviral vector was used to design third-generation CAR-NK cells (CD28.BB), which successfully eradicated CD4-expressing AML cells in vitro and demonstrated robust antileukemic activity in a CD4+ AML xenograft mouse model [173]. Besides, the first-in-human clinical trial used CD33 as a therapeutic target, which is expressed on both healthy and malignant myeloid cells, to develop a third-generation CAR lentiviral designed to reprogramme NK92 cells. In a GMP facility, CD33 CAR-NK cells were expanded. Infusion of CD33 CAR-NK cells was shown to be safe in this phase I/II study, but no reaction to treatment was seen in patients with acute myeloid leukemia (AML) (NCT02892695 and NCT02944162).

### CAR-NK cells in lymphoma

Non-Hodgkin’s lymphomas (NHLs) are a heterogeneous group of lymphoproliferative malignancies that originate in B-, T-, or natural killer (NK) lymphocytes. One of the appealing molecules to target B cell non-Hodgkin lymphoma (B-NHL) is CD20. Rituximab, a chimeric anti-CD20 antibody, is effective in treating mature B-NHL. However, certain B cell cancers are vulnerable to it or resistant to it. Chu et al. successfully modified and expanded peripheral blood NK cells ex vivo following anti-CD20 CAR mRNA nucleofection and demonstrated that anti-CD20 CAR-NK cells improved dramatically cytotoxicity against CD20+ B-NHL cells in vitro, including both rituximab-sensitive and rituximab-resistant B-NHL cells, and prolonged survival and reduced cancer size in xenotransplant (NSG) mice [160]. Peripheral T cell lymphomas (PTCLs) are a type of non-lymphoma Hodgkin’s (NHL) that comes up from mature T cells that express CD4 antigen widely and uniformly. As a result, CD4 may be an ideal candidate for CAR immunotherapy [178]. Pinz et al. developed the CD4CAR, a third-generation CD4-specific CAR with a single variable Fc chain in the extracellular domain for CD4 antigen recognition and signaling domains of CD28, 4-1BB, and CD3. CD4CAR-NK-92 cells were found to be directly and ruthlessly destroyed. CD4+ human T cell leukemia and lymphoma cell lines from adults and children, as well as patient samples, were studied ex vivo. The use of a third-generation CAR structure prolonged the lifespan of CAR-NK cells in vivo substantially suppressed lymphoma cell proliferation and greatly improved the survival of a xenogeneic mouse model [165]. Furthermore, it was discovered that anti-CD20-CAR mRNA electroporated PB NK cells and romidepsin had synergistic cytotoxic activities in both in vitro and in vivo one of mature B cell (CD20+) non-Hodgkin lymphoma (B-NHL) type, including Burkitt lymphoma (BL). Since romidepsin can significantly increase anti-CD20 CAR-expanded peripheral blood NK cells (exPBNK) in vitro cytotoxicity through NKG2D by inducing the expression of NKG2D ligands MICA/B in both rituximab-sensitive and rituximab-resistant BL cells [161]. Besides that, in another study, CD20-specific CAR-NK-92 injected directly into subcutaneous Daudi (Burkitt’s lymphoma) lymphoma xenografts suppressed local tumor development more effectively than CD19-specific CAR-NK-92 [158]., and in a subcutaneous Raji Burkitt’s lymphoma xenograft in a mouse model, CAR-modified CIK cells revealed potent antitumor activity and remarkably controlled disease development [155].

### Role of CAR-NK cells against multiple myeloma

Multiple myeloma (MM) is a malignant neoplasm of plasma cells that is characterized by clonal expansion of plasma cells in the bone marrow and the presence of monoclonal antibodies in the serum or urine. Over the last decade, treatment and prognosis methods for multiple myeloma have advanced dramatically. Since MM cells are extremely heterogeneous and the surface antigen for MM stem cells is unclear, finding an appropriate therapeutic target is a critical component of using CAR-NK cells to treat MM [179, 180]. The cell-surface glycoprotein CS1 (also defined as CD319, CRACC, and SLAMF7) is expressed in leukocytes, NK cells, and normal plasma cells. However, its expression was upregulated in MM, suggesting that c-Maf-mediated interaction with bone marrow stromal cells is responsible for MM cell adhesion, unrestricted proliferation, and tumorigenicity [181, 182]. New evidence shows that CS1 may be a potential target for CAR design. In this regard, a lentiviral construct of a CS1-specific CAR with a CD28-CD3z costimulatory signaling domain was designed and transduced into the NK-92 and NKL NK cell lines and their anti-MM functions were evaluated in vitro and an in vivo xenograft murine model of MM. The findings showed that CS1-CAR-NK cells could effectively eliminate CS1+ MM cells in vitro by enhancing MM cell removal and IFN secretion [166]. Furthermore, adoptive transfer of NK-92 cells expressing CS1-CAR directly reduced the growth of IM9 MM cells and resulted in long-term survival in aggressive MM xenograft murine models [166].

CD138 (syndecan-1) is an integral membrane protein and an early diagnostic antigen of MM cells that mediates MM development and proliferation, in addition to CS1. CD138’s upregulated expression on MM cells suggests that it may be a good choice for CAR immunotherapy. Jiang et al. used a lentiviral vector containing a CD138-specific CAR fused to the CD3z chain as a signaling domain to modify NK-92MI cells. When compared to the empty vector-transduced NK-92MI cells, these reprogrammed NK-92MI cells showed significantly increased CD107a expression and production of granzyme B, IFN- against CD138-positive human MM cell lines (RPMI8226, U266, and NCI-H929). In the xenograft NOD-SCID mouse model, irradiation of NK-92 cells was shown to block further cell proliferation while also exerting specific antitumor activity [167]. Since the current CAR manufacturing strategies include the formation of a specialized antibody domain to recognize cancer cells by CAR-engineered cells. It is likely, though, that using this technique would result in the loss of tumor antigen. In this case, primary NK cells from MM patients were expanded and transduced with a novel type of CAR include natural killer group 2 member D (NKG2D) CARs carrying 4-1BB and CD3z signaling domains showing considerably cytotoxic activity against the majority of MM cell lines. These findings pave the way for further research and development of this novel CAR build as a treatment option for aggressive MM [168].

## CRISPR-Cas9 application in CAR-NK cell-based therapies

Clustered regularly interspaced short palindromic repeats (CRISPR)/ CRISPR-associated protein 9 (Cas9) has recently developed as an encouraging technology for genome edition [183]. This technique depends on the insertion of Cas9 protein in association with guide RNA into the NK cells [184]. It can be utilized to exactly delete, repair, or insert genes in a particular locus and thereby hold promise to establish more effective antitumor NK cells. For the first time, this strategy was exploited in primary NK cells to impair the CD38 gene for avoiding the fratricide of NK cells when these cells were utilized concomitant with daratumumab (anti-CD38), as CD38 is typically expressed not only on NK cells but also on MM and AML cells [185]. It has been suggested that this technique could result in manufacturing more potent and persistent CAR-NK cells. For example, the use of homology-directed repair (HDR) templates improved the knock-in efficiencies about 75% using K562-IL-21-expanded NK cells [186]. As well, CAR expression induced by an endogenous promotor following CRISPR/Cas9-specific locus knock-in methods leads to the ameliorated and prolonged CAR expression in redirected effector cells in vivo, with a memory-like phenotype and less expression of exhaustion markers [187]. Further, Daher et al. found that CRISPR/Cas9-mediated knockout of cytokine-inducible SH2-containing protein (CIS) as a negative regulator of IL-15 signaling improved CAR-NK cell activity in vitro and in animal models partially by augmented aerobic glycolysis [188].

## Conclusions

Extensive research over the last few decades has shown the safety and credibility of using NK cells to treat cancer. However, NK cell therapy is still vulnerable to immunosuppressive mechanisms. To resolve immunosuppression, improve cancer cell targeting, and finally enhance the antitumor effects of NK cells in cancer immunotherapy, acceptable and effective gene manipulation systems are required. Although the CAR-NK cells have shown great promise as a new strategy in adoptive immunotherapy with effective anticancer function against refractory malignancies in various clinical trials (Table 3), but the architecture of CAR-NK cells yet requires further investigations. It is important to choose the opposite target and consider which NK cell source and which kind of CAR construct can improve the efficiency of CAR-NK cells. Besides, the components and regimen of the culture medium that are suitable for NK cells from various sources, improvement of NK cell expansion, and then manufacturing CAR-NK cells with memory properties in vivo for tumor surveillance remain to be determined. Moreover, the long-term anticancer efficiency following administration to patients is still unclear. CRISPR/Cas9-based genetic modifications are noteworthy innovations that might increase the cytotoxicity efficiency and safety of CAR-NK cells by specifically editing primary NK cell genes and constructing stably transduced NK cells. Nonetheless, considering advanced efforts to resolve remaining obstacles, as well as the rapid developments achieved by robust preclinical investigation and clinical research, CAR-NK cell products are expected to make a strong and important contribution in cancer therapy.

**Table 3:** Clinical trials based on CAR-NK cell therapy for human hematological malignancies registered in ClinicalTrials.gov (May 2021)

*Condition*	*Dose*	*Target Ag*	*Phase*	*Participant number*	*Location*	*NCT number*
Non-Hodgkin lymphoma (NHL)	2 × 10^6^ /kg 6 × 10^6^ /kg 2 × 10^7^/kg	CD19	Early 1	9	China	NCT04639739
B cell lymphoma (BCL)	50 × 10^3^ /kg 600 × 10^3^ /kg	CD22	Early 1	9	China	NCT03692767
Non-Hodgkin lymphoma (NHL	N.A	CD19	1	25	China	NCT04887012
B cell lymphoma (BCL)	50 × 10^3^ /kg 600 × 10^3^ /kg	CD19	Early 1	9	China	NCT03690310
Multiple Myeloma (MM)	N.A	BCMA	1/2	23	China	NCT03940833
B cell lymphoma (BCL)	50 × 10^3^ /kg 600 × 10^3^ /kg	CD19 CD22	Early 1	10	China	NCT03824964
Mantle cell lymphoma (MCL) Diffuse large B cell lymphoma (DLBCL) Non-Hodgkin lymphoma (NHL) Follicular lymphoma (FL)	N.A	CD19	1/2	0	USA	NCT03579927

## Data Availability

Not applicable

## Abbreviations

CAR: Chimeric antigen receptor
GvHD: Graft-versus-host disease
CAR-NK: CAR-transduced NK cells
NK cells: Natural killer cells
ICIs: Immune checkpoint inhibitors
ACTs: Adaptive cell therapies
NHL: Non-Hodgkin lymphomas
DLBCL: Diffused large B cell lymphoma
FL: Follicular lymphoma
ALL: Lymphoblastic leukemia
MM: Multiple myeloma
AML: Acute myeloid leukemia
CRS: Cytokine release syndrome
CTLs: Cytotoxic T lymphocytes
NCRs: Cytotoxicity receptors
SHP-1: SH-2 containing protein tyrosine phosphatase
ITIMs: Immunoreceptor tyrosine-based inhibitory motifs
hESCs: Human embryonic stem cells
SCGM: Stem cell growth medium
b-NHL: B cell non-Hodgkin’s lymphoma
RCC: Renal cell carcinoma
CAIX: Carbonic anhydrase IX
EpCAM: Epithelial cell adhesion molecule
iCAS-9: Inducible caspase 9
